# Exploring how researchers consider nutrition trial design and participant adherence: a theory-based analysis

**DOI:** 10.3389/fnut.2024.1457708

**Published:** 2024-12-17

**Authors:** Anna Worthington, Taylor Coffey, Katie Gillies, Rajshri Roy, Andrea Braakhuis

**Affiliations:** ^1^Discipline of Nutrition, Faculty of Medical and Health Sciences, The University of Auckland, Auckland, New Zealand; ^2^Health Services Research Unit, Health Sciences Building, University of Aberdeen, Aberdeen, United Kingdom; ^3^Nutrition and Dietetics, Sydney School of Nursing, Faculty of Medicine and Health, The University of Sydney, Sydney, NSW, Australia

**Keywords:** patient adherence, nutrition trials, behavior change science, methods, research design, treatment adherence and compliance

## Abstract

**Introduction:**

Nutrition trials are important for informing dietary and clinical guidelines. Central to the success of these trials is participant adherence to dietary behaviors. However, trials commonly experience poor adherence. This study seeks to understand if and how researchers consider supporting participant adherence to dietary behaviors and their relationship to using behavior change science when designing trials.

**Methods:**

A mapping exercise was undertaken to create matrices that describe the landscape of current nutrition trials. A total of 12 researchers participated in semi-structured, one-on-one interviews. Transcripts were analyzed using (i) the theoretical domains framework (TDF) to identify themes in current practice and beliefs, and (ii) the capability, opportunity, motivation, and behavior model to identify barriers and enablers to using behavior change science in the design of nutrition trials.

**Results:**

Twenty-two belief statements were identified across all 14 TDF domains and were conceptualized as 5 key themes with respect to designing nutrition trials to improve participant adherence: (i) what was done, (ii) how it was done, (iii) why it was done, (iv) adherence challenges, and (v) conflicting beliefs. Regarding using behavior change science when designing trials, some researchers felt this would be beneficial but lacked the knowledge and skills to do so, while others were skeptical of its value over the current experience-based practice.

**Discussion:**

Researchers are motivated to encourage participant adherence to dietary behaviors, and, consciously and subconsciously, implement a range of strategies through non-systematic methods in their trials. Future publications would benefit from the explicit documentation of levels of adherence to dietary behaviors and strategies implemented to improve adherence.

## Introduction

Nutrition intervention trials are key for informing dietary guidelines and clinical practices (1). Inherent to these trials, is the need for participants to perform certain dietary behaviors to answer questions about primary outcomes. Dietary behavior is an umbrella term that refers to all phenomena related to food choice, eating behavior, and dietary intake/nutrition (2). These can range from simple, such as taking a supplement or single food item, to more complex behaviors, such as changing whole dietary patterns. Hence, adherence to the dietary behavior, or the extent to which a participant actively follows an investigator’s instructions on the behavior, is often a spectrum rather than binary; additionally, it may be part of the intervention arm only or part of both intervention and control arm, i.e., a trial process. Given the challenging nature of changing one’s dietary habits, nutrition trials face a unique challenge in measuring and achieving adherence (3, 4). From here on, participant adherence specifically refers to adherence to dietary behaviors within a trial.

Commonly in trials, dietary behavior change is conceptualized as part of the intervention, where only participants in the intervention arm are asked to perform the target behavior. For example, participants in the intervention arm of a trial that looked at the metabolic effect of an adapted Mediterranean diet were asked to change their dietary pattern for 12 weeks, while the control arm was asked to continue their habitual diet (5). However, it is important to note that performing dietary behaviors may also be part of trial processes, whereby participants on all arms of a trial are required to perform that behavior. For example, in a study investigating the health effects of regular consumption of red meat compared to plant-based meat alternatives in young adults, both arms were required to adhere to a basal vegetarian diet (6). Indeed, a trial may involve dietary behaviors as part of the intervention and the trial processes. For instance, one crossover trial investigating kiwifruit consumption on intestinal function required participants to consume two kiwifruits daily for 3 days as the intervention arm, consume two isocaloric controls twice daily for 3 days as the control, and fast overnight prior to the MRI scan as part of the trial processes (7). Consequently, researchers may need to carefully design their trials to support participant adherence to multiple dietary behaviors of varying complexity and duration.

Nutrition trials exist on a continuum between efficacy trials, where adherence to a dietary behavior is required to elucidate the effect a food or dietary pattern has on human health, to effectiveness trials, where dietary behavior change is desired to understand its effect in a real-world setting (8). Central to the success of trials that lie closer to the efficacy end of the spectrum, is the need for participants to adhere to the dietary behavior change required within the trial (9, 10). Unfortunately, many nutrition trials suffer from low participant adherence and high attrition rates (7–10). For efficacy trials, the magnitude, or observed effect, of the dietary intervention on the primary outcome is dependent on the level of adherence. Poor adherence decreases the likelihood that the results reflect the true effect of the intervention (11). Measuring and reporting adherence to dietary behavior in both efficacy and effectiveness trials is essential for understanding its true influence on primary outcomes (12). However, there is often heterogeneous and insufficient documentation of adherence in nutrition trials (13–15). Consequently, it is important to support and measure participant adherence to dietary behaviors, as well as adequately report these efforts and their outcomes.

The design of a trial can either facilitate or hinder adherence (16). For instance, using behavioral strategies, such as goal setting and self-monitoring, has been shown to improve adherence to lifestyle interventions (17). When considered through the lens of behavior change science, these strategies are known as behavior change techniques (BCTs) and are defined as the “active ingredient” that brings about behavior change (18, 19). Despite research advocating for transparent and replicable methodology, little is documented about how or why researchers select and implement certain BCTs to enhance adherence to dietary behaviors, whether that be for the intervention or trial processes. Behavior change frameworks provide a systematic, theory-based way of selecting BCTs that are most likely to bring about change. Over recent years, these frameworks have been applied to improve the design of interventions aimed at changing health and environmental behaviors and are returning promising results in terms of efficacy (20, 21). Additionally, public health guidelines advocate for the use of such frameworks within strategy design (22). Consequently, using behavior change science, such as frameworks to select BCTs, not only in the design of lifestyle interventions but within the design of trials involving any dietary behavior, is likely a promising avenue to enhance participant adherence.

Given the breadth of nutrition trials, an important starting point for this research is to understand current trends in the types of nutrition trials being conducted, including conditions of interest, the complexity of interventions, levels of adherence, and the type of dietary behaviors trials involved. Additionally, this will identify a representative pool of researchers as potential participants across nutrition trials to interview. As such, the primary aim of this research is to understand the behavioral factors that drive nutrition researchers’ selection of strategies within the trial design to enhance participant adherence to dietary behaviors. Additionally, these researchers’ relationship to using behavior change science when designing trials involving dietary behavior changes will be investigated, and strategies to support future researchers in using behavior change science will be considered.

## Objectives

The purpose of this research is threefold:

- To describe the current landscape of nutrition intervention trials, including types of trials being conducted, conditions of interest, the complexity of interventions, and the types of dietary behaviors involved, and to identify the levels of adherence within these.
- To identify behavioral determinants that influence how researchers design nutrition trial components to support participant adherence to dietary behaviors.
- To understand researchers’ relationships with behavior change science, as well as the barriers and enablers to its use in the design of nutrition trials.

## Methods

The Consolidated Criteria for Reporting Qualitative Research (COREQ) checklist (23) was used to guide study reporting (Supplementary file S1).

### Part 1. Conceptualizing nutrition trial areas

As the field of nutrition research is so broad, a mapping exercise was undertaken to identify areas of high research activity within it. The Clinical Trials Registry (clinicaltrials.gov) was searched for nutrition intervention trials that had results first posted in the past year (1 June 2022–1 June 2023). A limited date range was selected to retrieve a manageable sample size. For ‘Other Terms’, “nutrition OR diet” was entered. Filters included selecting trials classified as interventions, conducted among adults (18+ years), that accepted healthy volunteers, and with recruitment marked as completed. A matrix was created that described the type of dietary intervention and condition investigated. Definitions of intervention types were as follows:

Simple intervention: trials in which the primary purpose is to investigate a supplement, drug, or single nutrient.

Dietary intervention: trials in which the primary purpose is to investigate a specific dietary pattern or combination of dietary components.

Multi-component intervention: trials in which the primary aim is also to change another behavior (e.g., physical activity) alongside diet.

Device and/or procedure intervention: trials in which the primary purpose is to investigate a device or medical procedure (e.g., gastric band).

A second matrix was created that described the type of dietary behavior in the trial (e.g., simple or complex) and the reported level of adherence. In addition, the incorporation of dietary behavior change into nutrition trials was considered by authors as desired or required. In trials investigating the effect of complex interventions, dietary behavior change is often desired of participants as a direct outcome of the intervention. For example, an intervention evaluating the effectiveness of a multi-component, community-based healthy eating program aims to encourage participants to change their dietary behavior as a result of the intervention. On the other hand, trials evaluating the physiological or psychological effect of consuming a specific supplement, nutrient, food, or dietary pattern require participants to change their behavior. For example, an efficacy trial investigating the impact of a low-fat diet on bile production requires participants to adhere to a low-fat diet to elucidate the primary outcome of the study.

Data for these matrices were extracted from the information published on clinicaltrials.gov, attached files (e.g., protocols), and associated publications. Of note, not all trials from the search had published articles with relevant data on adherence and as such were coded as ‘data not available’ in the second matrix.

### Part 2. Interviews

#### Sampling and recruitment

All corresponding authors from the articles identified in Part 1 were contacted via email inviting them and/or their co-authors involved in the trial design to participate in one-on-one interviews; this convenience sample was deemed reflective of a range of articles reporting high, low, and insufficiently reported adherence to dietary behaviors. Upon expression of interest following this email, they were sent a second email with an attached participant information sheet (PIS) and consent form. Potential participants were told author AW would be the interviewer and provided brief information on her research in the PIS; no other interviewer characteristics were provided. Consenting authors of articles will hereon be referred to as ‘researchers’.

Previous literature has recommended that a minimum of 10 interviews be conducted for initial data analysis, followed by three additional interviews until data saturation is reached, that is, there are three consecutive interviews where no new themes arise (24). Hence, to identify when this stopping criterion was reached, data were analyzed concurrently with progressive collection.

#### Data collection

Semi-structured qualitative interviews were conducted, and video was recorded on Zoom (version 5.16.10, Zoom Video Communications Inc.) between 29 August 2023 and 23 November 2023. An interview topic guide was developed using the Theoretical Domains Framework (TDF) and the Capability, Opportunity, Motivation, Behavior (COM-B) model (18). The topic guide was refined by a discussion between two researchers experienced in using the TDF and COM-B models following mock pilot interviews. The final version can be found in Supplementary file S2. There were two main parts to the interview. The first part aimed to understand the determinants that influence how researchers design nutrition trial components to support participant adherence to dietary behaviors. The second part aimed to understand researchers’ relationship to behavior change science, and their barriers and enablers to using it in nutrition trial design.

All one-on-one interviews were conducted by AW who is a New Zealand Registered Dietitian; at the time of the interviews, she was a full-time PhD student and had 3 years of experience in running focus groups and one-on-one interviews, as well as training in using the Behavior Change Wheel, the TDF, and BCT taxonomy version 1 (BCTTv1). The interviewer adapted the order of questions within the topic guide to facilitate the natural flow of conversation and took field notes throughout. The interviews lasted up to 60 min.

#### Data analysis

Audio transcripts were exported using the Zoom software and then checked by AW that they had been transcribed verbatim. Identifying information, such as names and organizations, was removed. Transcribed interviews were sent to researchers within 1 week for them to amend or withdraw parts of the transcript; at this stage, they were provided the opportunity to add further written comments at the end of the transcript. Researchers were given a 2-week timeframe for this task from the date the email was sent; they did not provide feedback on the overall findings.

Returned transcripts were uploaded to NVivo (Version 12) for theory-based content analysis. A coding guideline (i.e., a set of explicit statements of how the TDF is to be applied to a specific data set) was developed by AW and TC (Supplementary file S3) (25). This was used by AW to deductively code statements into the most relevant TDF domain. A second author (TC) independently coded two of the first four transcripts, and discrepancies between coders were discussed to iterate the interview script and coding guideline to ensure that all relevant data were being captured and accurately coded. Specific belief statements were then inductively generated within each theoretical domain by AW; belief statements are statements that summarize a collection of responses with a similar underlying belief influencing the target behavior (26). Where possible, frequency counts of certain belief statements across all interviews were generated by counting once within each interview. Relevant theoretical domains were identified by the (1) relatively high frequency of specific beliefs, (2) presence of conflicting beliefs, and (3) evidence of strong beliefs that may affect the target behavior (27). Additionally, the BCTTv1 was used to classify strategies researchers reported using to enhance participant adherence (19); each BCT was recorded only once across all transcripts to demonstrate the range of techniques used as opposed to the frequency.

## Results

### Part 1. Conceptualizing nutrition trial areas

The search returned 55 registered trials. For the purpose of this study, the authors were interested in adherence to eating behaviors, and consequently, trials not involving an act of consumption were excluded (*n* = 7). Additionally, 11 studies were excluded as they focussed on other topics (e.g., smoking cessation and physical activity). The remaining 37 trials were categorized by the intervention type [i.e., simple, dietary, complex, or device (Table 1)] and health condition related to the primary outcome.

**Table 1:** Matrix of nutrition trials with results published on clinicaltrials.gov between June 2022 and June 2023.

Primary intervention type Condition^&^	Simple	Dietary	Multi-component interventions	Device	Total studies
Obesity	B* X* X* X* X* X	X* X X	X X	X	12
Healthy (establishing food safety, biomarker, etc.)	X* X* X		B		4
Osteoarthritis/osteoporosis			X X		2
Pregnancy		X	X X		3
Cognitive function	X* X				2
Cardiovascular disease	X		B X		3
Diabetes Mellitus	X	X	B B X		5
Gastrointestinal health	X* X* X	X			4
Other (aging, cancer)			B* X		2
Total Studies	16	6	14	1	37

B, behavioral primary outcome; X, physiological primary outcome; *, Intervention ≤ 4 weeks.

^&The condition of the trial’s primary outcome focussed on preventing, improving, or understanding.^

**Definitions of intervention types:**

**Simple intervention: trials in which the primary purpose is to investigate a supplement, drug, or single nutrient.**

**Dietary intervention: trials in which the primary purpose is to investigate a specific dietary pattern.**

**Multi-component** intervention: trials in which the primary aim is also to change another behavior (e.g., physical activity) alongside diet.

**Device and/or procedure** intervention: trials in which the primary purpose is to investigate a device or medical procedure (e.g., gastric band).

Table 1 shows a high proportion of nutrition trials had a primary outcome related to obesity (*n* = 12). In terms of intervention type, there were a similar number of simple (*n* = 16) and complex interventions (*n* = 14) reported. The majority of trials had a primary outcome that was physiological (*n* = 31) rather than behavioral. Complex interventions tended to be longer than 4 weeks, while simple interventions were shorter in duration.

Table 2 shows that, of the studies that had data available, the majority (*n* = 15) did not report adherence in sufficient detail, if at all. In trials that required behavior change, there was a mix of simple and complex dietary behaviors; of those with high adherence, all intervention supplements or food were provided. Of the identified studies, all desired behaviors were complex, did not provide the totality of food, and half had insufficient or no adherence to the behavior reported.

**Table 2:** Adherence of nutrition trials with results published on clinicaltrials.gov between June 2022 and June 2023.

Dietary behavior Adherence	Required	Desired	Total studies
Higher (>80%) or statistically significant change	S* S* S* S* S* C* C*	C C	9
Lower (<80%) or no statistically significant change		C C C	3
Not reported in sufficient detail	S* C	C C C C C C	8
Not reported within the article	S* S* S C C C	C	7
Data not available^&^	S* S* S* S* S* C* C* C*	C C	10
Total studies	23	14	37

*All intervention supplements or food provided.

^&Results of identified trials in the registry were not published at the time of the search.^

S—Simple dietary behavior: behavior that requires minimal steps, e.g., taking a pill, supplement, or performing a one-off behavior.

C—Complex dietary behavior: a behavior that is a combination of different behaviors, e.g., changing multiple dietary behaviors to follow a dietary pattern.

Required—behavior participants need to perform within a trial to maintain validity.

Desired—behavior desired of participants as a direct outcome of the intervention.

### Part 2. Interviews

#### Participant characteristics

Twelve researchers responded and consented to participate (50% female), while no response was received from the other researchers. The majority of researchers worked in the United States (*n* = 9), while the remaining individuals worked in Italy, the United Kingdom, and New Zealand. The majority of researchers (*n* = 8) were principal investigators of the trial they were identified through; other roles included research dietitians (*n* = 2), a study coordinator (*n* = 1), and a principal scientist (*n* = 1). All researchers were involved in the design and conduct of the trial described in the publication they were identified through. Researchers reported having between 5 and 45 years of research experience, with an average of 18 years. Two researchers considered themselves experts in behavior change science. With this sample size, it was deemed that sufficient depth of understanding of the phenomenon had been reached.

#### Interview part 1. How do researchers design nutrition trials to support participant adherence?

Twenty-two belief statements were identified, covering all 14 TDF domains, and can be conceptualized as 5 key themes with respect to designing nutrition trials to improve participant adherence to dietary behaviors. The themes are (i) what was done, (ii) how it was done, (iii) why it was done, (iv) adherence challenges, and (v) conflicting beliefs (Table 3). TDF domains are included below in parentheses for further context.

**Table 3:** Determinants that influence how researchers design nutrition trials to support participant adherence to dietary behaviors.

Theme	Belief Statement (*theoretical domain*)	Illustrative quote
What was done	We defined and attempted to measure adherence to dietary behaviors in our trial (*knowledge*)	*“… we had to understand if they were adherent to the different diets, one which was a Mediterranean diet, another which was a lacto-ovo vegetarian diet. So we tried to estimate the adherence with two measures. One was the questionnaire…and we performed a 24 [hour] recall…” P5*
	We have implemented some strategies to try and improve adherence in our trial *(skills)*	*“So we actually gave our participants things like a large jug of olive oil. We collaborated with certain brands that provided walnuts or almonds, things like that. So, we tried to reinforce, you know, a diet that has a lot of evidence behind it” P4*
	When recruiting, we screen for people we think are more likely to be adherent (*skills*)	*“When you enroll people it’s [important] to really make sure you get people who fully understand and are, I guess you could say fully, or are motivated. So find people who are motivated and want to change and want to do this study and want to want to follow my instructions.” P8*
	We made compromises in the trial design to support participant adherence, as a good trial design can improve participant adherence (*beliefs about consequences*)	*“We kind of sacrificed this piece of it like controlling what really they are having because we thought that this would increase the feasibility and increase the generalisability of the study, you know. So, I think that for nutrition trials in particular, I think it’s pretty tough to have a trial that is so controlled, because that’s where I think adherence becomes more complicated.” P3*
How it was done	Reflecting on my experience in trials helps me choose what strategies to use to support participant adherence (*behavioral regulation*)	*“So I was designing and doing different studies, and trial and error… So I think I would credit that to my postdoc and the ability to run all kinds of different studies and see what’s worked.” P8*
	We use the literature to inform what strategies we choose to improve participant adherence (*memory, attention, and decision processes*)	*“We try to understand what was has been done in the literature our best. We had some meetings before in order to understand how could we improve the adherence, and we found these strategies in the literature.” P5*
	We consider what would stop participants from adhering when designing our trial (*memory, attention, and decision processes*)	*“We really tried to think about all the places that people make decisions about food and have something in place for every single one of those.” P7*
	I seek advice from other members of my team, participants, or experts to help choose strategies that are more likely to improve adherence (*social influences*)	*“We discuss together when we decide to design study also for this [adherence] aspect. We have, we are a multidisciplinary team, so some are more involved in this aspect, some others less. We are all susceptible to this problem and we try to find a solution we can, we can do in this context.” P5*
Why it was done	I am highly motivated to encourage participant adherence (*intention*)	*“[I’m] highly motivated, because it’s actually quite a job getting participants to commit to something like that, and we do not want to have too many dropping out, or have to recruit large number to allow for that. So, it’s pretty important the ones we get will stick with it, and that we do not need to allow for too many dropouts.” P12*
	When designing a trial, it is part of my role to help participants be adherent (*social, professional role, and identity*)	*“I think that it’s my role to really worry about how I’m going to get people to enroll and then to stay.” P3*
	Poor adherence can mean we have to spend more time and money on our trial to make it work, so we try to think about it from the start (*reinforcement*)	*“Someone who’s not adherent will potentially skew your results. Potentially, it means that you have to recruit more people and spend more money or spend more time or resources. So… that’s the incentive to think about it in advance, and then come up with good strategies to make sure people adhere, because if they do not, your study might just not work.” P11*
	Our choice of strategies to support adherence is influenced by our resources (budget, personnel, and time) (*environmental context and resources*)	*Interviewer: “Do you recall how you decided what strategies to use to improve participant adherence?”* *P10: “Usually it’s budgetary, unfortunately…”* *“… It’s one of those things that we have to be available when the participant needs you. But I think that’s actually a big challenge to be available when people needed us, because we did not have the infrastructure where we could just always be on call.” P3* *“…if they had all the money in the world they would use the best methods available as well, but sometimes, you know, the diet intervention methods they slowly get kind of chipped away in order to fit into the budget.” P7*
Adherence challenges	Trial participants are only human and this can make it hard to achieve adherence (*social influences*)	*“The whole idea of adherence is impossible. And then a dietary intervention is impossible. I mean, they are not lab animals. So you just gotta have expectations. And I think granting agencies do not always realize that. And even reviewers that you need to be realistic…these are humans who go to work and parties in school and high school, and whatever grade they are in. And you know they are gonna eat things they are around that you do not want them to eat or they are not gonna keep track.” P2* *“And knowing that participants are human and that they have their lives, you know, participating in studies is not their primary goal, maybe probably even secondary or tertiary and so working within those confines.” P1*
	Measuring dietary behaviors to assess adherence is flawed and difficult *(beliefs about consequences)*	*“So we look at adherence more as session attendance or self-monitoring adherence. But we know that dietary measurement is so messy and so prone to errors that it does not seem like the ideal focus.” P1* *“…it’s all self-report so I have no way of to know if they really took it, but they seem to be.” P2*
	My confidence to run a study with high adherence depends on the complexity of the dietary behavior (*beliefs about capabilities*)	*“I think it depends on how complicated your dietary intervention is… If you are trying to change a whole diet. and for a prolonged period, you know longer than for a couple of days, I imagine that would that that’s not something I’ve had, I’ve done, but I think that that would be extremely challenging, and I’m not sure that I would have huge amount of confidence doing that level of nutritional or dietary intervention” P11*
	Promoting adherence can be stressful or frustrating for me (*emotion*)	*“I get feelings of a little bit of stress, because I remember how challenging it was.” P4*
Conflicting beliefs	Achieving good participant adherence is important, but recruitment or achieving the outcome takes priority (*goals*) vs. Achieving good participant adherence is one of the highest priorities for us (*goals*)	*“The goal was weight management, and so our supervision was more focused on were they meeting the expected weight trajectory rather than were they meeting our standards related to dietary adherence.” P1* vs. *It’s one of the highest priorities… if our participants are not adhering to the diets, my job is pointless, you know. So it’s high priority. P7*
	I was hopeful participants would adhere, but not widely optimistic they would (*optimism*) vs. I expected participants to adhere to the desired dietary behavior because we made it easy for them (*optimism*)	*“We’re not forcing them to be in the trial. They know what they are getting into. So, I mean, I do not know if I would say I’m widely optimistic, but I think it should work.” P2* vs. *“Yes, I expected them to, because it wasn’t a particularly difficult ask and we had quite clear instruction and it wasn’t over a prolonged period of time, so there was, there should not have been much to stop them from being able to adhere to it. We made it very simple for them.” P11*
	I do not give a lot of thought to participant adherence (*behavioral regulation*) vs. I think about participant adherence from the beginning of trial design (*behavioral regulation*)	*“It’s not something I’ve actually given a lot of thought to, participant adherence, even though it’s obviously very important.” P12* vs. *“I think you have to think out [participant adherence] ahead of time… you just have to do that extra work.” P6*

#### What was done?

The majority of researchers (*n* = 10) reported that dietary adherence was defined in their trial (knowledge), while one said it was not, and another could not remember. Nine participants also reported measuring dietary adherence within their trial. The method of measuring adherence was dependent on dietary behavior. Types of measurements included self-reported questionnaires specific to the study behavior, collection of containers or food waste to prove consumption, 24-h recalls, doubly labeled water, witnessing food being consumed (e.g., via Zoom or photos), and blood biomarkers. Many researchers believed that trial design impacts a participant’s ability to adhere (beliefs about consequences), and as such made compromises in the trial design to support participants. Additionally, many researchers highlighted that to improve adherence, they screened for people who were more likely to be adherent to their targeted dietary behavior when recruiting (skills). All researchers reported using strategies (skills) to enhance participant adherence that could be classified as BCTs. Using the BCTTv1 (19), 14 BCTs were identified across the transcripts (Supplementary file S4); few researchers described these strategies using BCT terminology.

#### How it was done?

Many researchers expressed how their experience in trials was primarily what informed trial design and their selection of strategies to support participant adherence (behavioral regulation). Additional aspects that informed chosen strategies to support participant adherence included using the literature and strategies used in similar trials (memory, attention, and decision processes), seeking advice from other members of their team or experts (social influences), and thinking about the potential participant barriers to the targeted dietary behavior (memory, attention, and decision processes). For some, thinking about supporting adherence was a conscious process, while for others, choosing strategies to support adherence was done implicitly.

#### Why it was done?

The majority of researchers (*n* = 10) saw it as part of their role to help participants be adherent (social professional role and identity) and felt highly motivated to achieve participant adherence (intention). Researchers saw it as important to encourage adherence, as poor adherence decreases the validity of the trial and wastes resources (reinforcement/beliefs about consequences). However, the study design and strategies chosen were influenced by the budget and time available for each trial (environmental context and resources).

#### Adherence challenges

One of the most common challenges voiced was difficulty achieving adherence due to trial participants “*just doing what they want to do*” (P2) (social influences). Additionally, researchers voiced accurately measuring dietary behaviors was a challenge due to believing existing measures of dietary assessment are flawed and difficult to conduct (beliefs about consequences). The complexity of the dietary behavior in question also impacted researchers’ confidence levels, with more complex behaviors lowering confidence in their ability to conduct a study with high adherence (beliefs about capabilities). Together, these challenges contributed to feelings of stress or frustration regarding adherence (emotion).

#### Conflicting beliefs

Three domains had the presence of conflicting belief statements. The majority of researchers voiced the importance of achieving good participant adherence. For some, it was one of the highest, if not the highest, priority, while for others, achieving the trial outcome or recruitment took priority (goals). Researchers also differed in their expectations of participants adhering; one group held an expectation that participants would adhere to dietary behaviors in their trial, while another group was hopeful but not certain they would adhere (optimism). Finally, researchers differed in how much they thought about participant adherence when designing their trial; some had developed a habit of thinking about it from the start of trial design, while others reported not giving it a lot of thought, despite recognizing its importance (behavioral regulation).

#### Interview part 2. Barriers and enablers to using behavior change science

Supporting quotes can be found in Supplementary file S5.

#### Capability

Many researchers expressed they do not have adequate knowledge about what behavior change science is, or how to use it in the design of nutrition trials. Confidence levels in their perceived ability to use behavior change science varied depending on their area of expertise and years of experience designing trials. For instance, dietitians with training in using behavior change science reported higher confidence levels. One behavior change expert expressed concern at other researchers using behavior change science when outside their area of expertise, describing it as “*contains a lot of subtleties that come from years of experience*.” Researchers suggested further training in applying behavior change science and having real-life examples to work from to increase confidence in their capability. Many were confident in their skills to do so if training was provided.

#### Opportunity

In general, researchers responded positively about the acceptance of using behavior change science among them and their colleagues. Two researchers were skeptical that it would be encouraged by other researchers and reviewers. Additionally, a key enabler identified through the interviews for using behavior change science was collaboration and networking with colleagues, particularly those who have more experience in its implementation. Discussing with colleagues was perceived as a way for researchers to question and improve current practices. Researchers perceived limited time and funding would stop them from using behavior change science in trial design. Access to resources, training, expertise, and literature was seen as enablers, as these were perceived to increase capability.

#### Motivation

Some researchers reported they would be motivated to use behavior change science if there was reinforcement from funding agencies. Equally, some researchers expressed how they had not thought about using it; it does not exist as a habit for them. About half of the researchers reported high motivation to use behavior change science in this context as they saw it as important for improving trial design and validity, and beneficial for the wellbeing of their participants. The other half of participants expressed lower motivation to use it, primarily due to the lack of evidence that it would lead to greater adherence than their current practices.

## Discussion

Given that nutrition trials often suffer from poor adherence to dietary behaviors, this study sought to understand if and how researchers consider participant adherence to dietary behaviors when designing a trial. The qualitative aspect of this study identified that many researchers consider participant adherence as important and attempt to define, measure, and support dietary adherence, often by relying on methods that have worked in their own experience or those used in other trials. When probed about using behavior change science in the design of trials, one group of researchers felt this would be beneficial but lacked the knowledge and skills to do so. Another group of researchers were more skeptical about the use of behavior change science in this way without evidence that it works better than current practice.

The matrices echo previous literature, highlighting the issue of poor participant adherence across different types of nutrition trials (1). In particular, they demonstrate that trials involving complex behaviors in real-world settings are more likely to have poor adherence than those involving simple behaviors or those where all food is provided (i.e., controlled feeding studies). Indeed, the confidence level of researchers in their ability to run a trial with good adherence decreased as the complexity of the behavior increased. All researchers reported thinking about participant adherence to various extents when designing their trial, and as a result, implemented strategies to support their participants. Few researchers had a systematic process for doing this, relying instead on their own experiences or those of others. This selection process, based on intuition, experience, and assumptions, is used by many when designing interventions to change health behaviors, including clinicians (28), and is known as the “*It Seemed Like A Good Idea At The Time*” (ISLAGIATT) principle (18). This is not to say this method is ineffective, but it certainly is not transparent or replicable, as advocated for in research (29). Together, this could indicate a need for researchers to use different methods of selecting strategies for enhancing adherence depending on the complexity of behavior in question. From this research it could be inferred using the ISLAGIATT principle is sufficient for informing the design of trials involving simple dietary behaviors, while a more systematic, evidence-based method may be more effective for supporting adherence to complex dietary behaviors.

Many researchers looked to other articles to see what strategies they used to support adherence. Consequently, sufficient reporting in articles about how adherence was supported, such standardized reporting using the BCTTv1 (19), and the resulting level of adherence, is important to build a greater understanding of how this can be improved. Of course, from the lens of behavior change science, care needs to be taken with this method; transplanting what works for one behavior in one specific context and population is not guaranteed to work in another (30, 31), which could perpetuate the problem of poor adherence and its consequences on research validity and resource waste.

This is where providing a systematic method to select evidence-based BCTs, such as using behavior change frameworks, could play a key role. However, although all researchers viewed it as part of their role to enhance participant adherence, how many go about this is currently a self-taught, experienced-based art, as opposed to a systematic science. In our interviews, there was a consensus of insufficient knowledge and skills to apply behavior change science in trial design. Possible enablers suggested by researchers, and also aligning with expert recommendations (30), included providing further training on how to do so, or including behavioral scientists on the trial team. Additionally, some researchers were unmotivated due to the absence of evidence demonstrating that the use of behavior change science in this way would enhance adherence more than current practices. Indeed, the evidence base supporting this practice is still in its infancy, although it shows promising results (30, 32, 33). It is also important to note that there is little empirical evidence on the effectiveness of efforts to support adherence using the ISLAGIATT method (34).

The matrices highlight a potentially more imminent problem though. Despite the majority of researchers saying they define and measure adherence, as well as iterating the importance of it, the matrices indicate there is often no or inadequate reporting of dietary adherence. Additional literature also highlights this issue (15, 35). One possible reason for this is the lack of emphasis on documenting adherence to current reporting guidelines such as CONSORT. Recently, a Nutrition Extension for CONSORT was proposed and peer-reviewed (36, 37). An important addition is the requirement of articles to report the level of dietary adherence, or compliance with the intervention, in the trial and discuss the implications of adherence within the trial (36). This addresses previous research that has advocated for nutrition trials and reviews to report the definition and assessment of adherence in the methods and the resulting degree of adherence in the results (15).

Considering general compliance with CONSORT reporting guidelines is poor (35, 38), researchers may need additional support to comply with the CONSORT Nutrition Extension guidelines. For instance, dietary adherence must be measured to be reported. Researchers may need more support with selecting and implementing methods of dietary assessment, as doing so was viewed as a challenging aspect of trial design by researchers involved in this study. Framing the use of the CONSORT Nutrition Extension as behavior and applying learning from implementation science could add value and may be of use to facilitate uptake.

Strengths of this research include its basis on the TDF and COM-B model; this provided a systematic and thorough framework to explore the current beliefs and behaviors of researchers, such as insight into how researchers make efforts to enhance participant adherence, something often not reported in the literature (35). Understanding current behaviors, what drives them, as well as barriers to using behavior change science, provides a foundation for designing support to change researchers’ behaviors. However, the sample size, although diverse, was small and subject to self-selection bias, limiting its generalisability to other settings and populations. It was not powered to identify differences in beliefs and behaviors by researcher characteristics, such as their years of experience or background training, although these undoubtedly influence how one approaches designing a trial. Another potential limitation is we did not compare what researchers said they did, with what was reported in their article.

## Conclusion

Researchers are motivated to encourage participant adherence and implement a range of strategies to do so, often through non-systematic methods. Some researchers perceived behavior change science to be a promising alternative to improve trial design, while others were skeptical of its value over current practice. To build the knowledge base of how participant adherence can be enhanced, future research would benefit from the explicit documentation of strategies implemented in nutrition trial design and the resulting level of adherence.

## Data Availability

The raw data supporting the conclusions of this article will be made available by the authors, without undue reservation.
