# Supplementary material for: Exploring how researchers consider nutrition trial design and participant adherence: a theory-based analysis
Source: Front Nutr. 2024 Dec 17;11:1457708. doi: 10.3389/fnut.2024.1457708 (PMC11685074; doi:10.3389/fnut.2024.1457708)
Supplement: Supplementary file 2 [file Supplementary_file_2.docx]

**Topic Guide – TDF Semi-structured interviews**

**Behaviour of interest:** how researchers design and conduct human trials to promote/enhance participant adherence to dietary behaviours required or desired within that trial.

**AACTT Specification^^[[1]](#footnote-2)^^**

- Action: Any action taken, implicitly or explicitly, by researchers to encourage participant adherence to dietary behaviours
- Actor: Researchers involved in the design of human trials that involve participants performing a dietary behaviour
- Context: Tertiary institute, workplaces and other settings that conduct human trials
- Target: Trial participants
- Time: During the design and conduct of human (nutrition) trials

**Aims**

**Part 1.** Understand the determinants that influence how researchers design nutrition trials components to support participant adherence to dietary behaviours.

**Part 2.** Understand researchers relationship to behaviour change science, and their barriers to using it in nutrition trial design.

**Definitions that may be needed throughout the interview**

**Behaviour:** “Anything a person does in response to internal or external events. Actions may be overt (motor or verbal) and directly measurable or, covert (activities not viewable but involving voluntary muscles) and indirectly measurable; behaviours are physical events that occur in the body and are controlled by the brain”^^[[2]](#footnote-3)^^.

**Behaviour change science:** The practice of using models, theories and frameworks to understand and change behaviour.

**Nutrition intervention trial:** any human trial involving a dietary behaviour change.

**Adherence:** the extent to which participants’ behaviour corresponds with the agreed recommendations (e.g. from the PIS/trial staff).

**Adherence behaviour:** a behaviour performed by participants that impacts the outcome of the study, such as following a dietary pattern for a certain length of time.

**Retention behaviour:** a behaviour performed by participants relating to the return of outcome data, such as attending clinic visits and completing questionnaires.

*Before the interview, the full article that the participant was identified through will be read to identify and record behaviours that were required to be established, changed or maintained during the study. These may be discussed in the interview.*

**Interview opening**

Thank you for agreeing to speak with me today. I’m Anna, a researcher at the University of Auckland, and I’ll be conducting this interview which should take around 1 hour. There are two parts to this interview. In the first part, I’d like to know about your views on how you might affect participant adherence within nutrition trials, and any strategies you may use to encourage adherence. In the second part, I will be exploring your thoughts on using behaviour change science in the design of nutrition trials. Please note, you are not expected to have a background in behaviour change science and I will explain terms as we go through the interview.

Feel free not to answer any questions and you can withdraw at any time. The transcript of this interview will be anonymised and you will not be specifically identifiable in published results, although, as discussed in the participant information sheet, your name may be included in the reference list as we will reference your article (if it has been published). Can I confirm, are you okay with this?

After the interview, you will be emailed your transcript to amend or change before it is analysed, which I’ll speak more to at the end. There may be some overlap in the questions, so it’s ok to repeat answers. I’ll also type notes during the interview. As per the consent form, this will be recorded for the purpose of analysis but the transcript of this interview will be anonymised. Are you okay with this?

-SWITCH ON RECORDER AND

-READ OUT PARTICIPANT NUMBER

***For the purpose of recording could you please confirm again that you are happy to be interviewed today and happy to record our conversations?***

There are no right or wrong answers and we’re not interested in making any judgements. The purpose of this is to hear about your views and experiences relating to participant adherence so that we can best help future trials. Do you have any questions before we start?

| **Construct (if relevant)** | **Question** | **Possible prompts** |
| --- | --- | --- |
| **PART I** | | |
| **Background about trial/staff** | First, I have some background questions.  How do you identify your gender?  What country do you work in?  What is your job title?  How many years of research experience in nutrition trials do you have? | What area of nutrition research are you predominantly involved with? |
| *For the purpose of this interview, I want us to focus on adherence to dietary behaviours within nutrition trials, or the extent to which participants stick to the dietary behaviour they are being asked to follow/do within a trial. When I say ‘dietary behaviour, I am referring to any behaviour involving the consumption of a food or food supplement. Do you have any questions about this?* | | |
| **Opening questions** | I’d love to talk a bit about your **[name of study]**. Can you please tell me what your role was in the design of this trial?  In this trial, was adherence defined? If so, how? | Can you please tell me about the population, intervention, comparator and primary outcome? (PICO elements)  Were the participants free-living (community-dwelling) or under controlled conditions? |
| **Knowledge** | Within this study, what were the dietary behaviours participants had to adhere to?  How well did participants adhere to **[described behaviours]** in your trial? | How was adherence measured within this trial? |
| **Skills** | Can you tell me about any strategies you implemented in the trial to encourage participant adherence to these behaviours? | Do you feel there are any particular skills that have helped you design/implement this trial that have had an impact on adherence? |
| **Memory, attention and decision processes** | Do you recall how you decided what strategies to use to improve participant adherence? | What other factors influenced your decision to use these strategies? |
| **Social/Professional Role and Identity** | When designing a trial, do you consider it part of your role to consider how to enhance participant adherence to dietary behaviours? | Who’s role is it? |
| **Beliefs about consequences** | How do you feel trial design impacts participant adherence? | What sort of impact do you think your role has to participant adherence? |
| **Beliefs about capabilities** | How confident are you that you can design/conduct a nutrition study with high adherence to dietary behaviours? | - ***If low:*** what made you less confident? Is there anything that would increase your confidence?  -***If high-*** What made you feel more confident? |
| **Optimism** | Overall, did you expect participants to adhere to **[described behaviour]** in your trial? | -Why were you ***so*** optimistic?  -Why were you ***not so*** optimistic? |
| **Reinforcement** | What encourages/discourages you to consider ways to improve participant adherence in trials? |  |
| **Intentions** | How motivated are you to have high participant adherence to dietary behaviours in trials? | What are the reasons for this high/low motivation? |
| **Goals** | Where does enhancing participant adherence fit in your priorities when designing and implementing nutrition trials? (given the many competing priorities) | What other things take priority? |
| **Environmental context and resources** | What barriers do you experience when it comes to trying to improve participant adherence?  Did you have the necessary resources to improve participant adherence? | Are there any outside influences that make your role more difficult/easier?  Do certain situations/contexts make it more difficult/easier? |
| **Behavioural regulation** | What do you think could help overcome these barriers? | Are there any ways of working that you find helpful when trying to improve adherence? (E.g., do you keep a diary or notes?) |
| **Social influences** | How does the team you work with influence your approach to considering participant adherence in trial design? | How do the trial participants influence your efforts to improve adherence?  Is your team supportive of your efforts to improve adherence? |
| **Emotion** | This may be bit of an odd question, but bear with me.  What sort of feelings come to mind when you think of participant adherence in the trial? | Can you tell me a bit more about why you think these feelings arise?  Is it ever stressful/frustrating to try and improve adherence? |
| *Thank you. Those are all the questions for part 1. Did you have any other comments you’d like to add? If not, would you like to take a couple of minute break before we continue? Thank you for your answers. I’d like to move onto part 2 now which will explore your views on behaviour change science. A few background questions to start.* | | |
| **PART II** | | |
| **Background questions to establish behaviour change expertise** | Would you consider yourself an expert in behaviour change? (Yes/No)  What does the term ‘behaviour change science’ mean to you? | For experts: How many years’ experience do you have using behaviour change science? Can you briefly describe what behaviour change models, theories or frameworks are you familiar with? |
| *I will use the term behaviour change science a fair bit from here. When I say behaviour change science I am referring to “The practice of using models, theories and frameworks to understand and change behaviour”.[Show PowerPoint] – “We are proposing that researchers could use behaviour change science to enhance participant adherence, in a similar way to how health care professionals are encouraged to use it to enhance patient adherence to medications.”*  What would stop researchers, like yourself, from using behaviour change science in this way?  What would enable researchers, like yourself, to use behaviour change science in this way?  Additional prompts: | | |
| **Capability (psychological)** | To what extent are you confident you have the knowledge to do this? |  |
| **Capability (physical)** | To what extent are you confident you have the skills to do this? | What additional training would you require? |
| **Opportunity (social)** | To what extent do you feel this would be accepted/supported by the people around you (e.g. colleagues, stakeholders)? |  |
| **Opportunity (physical)** | What resources would you need to use behaviour change science in this way? |  |
| **Motivation (automatic)** | Is there anything else that would encourage/discourage you/others from using it?  Do you have any specific worries/concerns about using it? |  |
| **Motivation (reflective)** | How motivated do you feel to use behaviour change science in the design of nutrition trials? | Why is that?  How difficult/easy would it be for you to do this? |
| **Closing remarks** | Do you have any other thoughts you’d like to share? |  |

Thank you so much for your time today. I really appreciate your participation. I will transcribe the interview and get it back to you over the next week. You’ll receive a word document of the transcript that you can edit to amend or withdraw any statements, as well as add any additional comments in a box at the end. Due to needing to analyse data concurrently with interviews, are you able to send me back your amended transcript within two weeks? I’ll send a reminder after 10 days, and if I don’t hear otherwise within 2 weeks I will use the transcript in the analysis as is. Do you have any questions? Again, thank you so much for your time today.

**Slides shown to participants at part II of interview:**


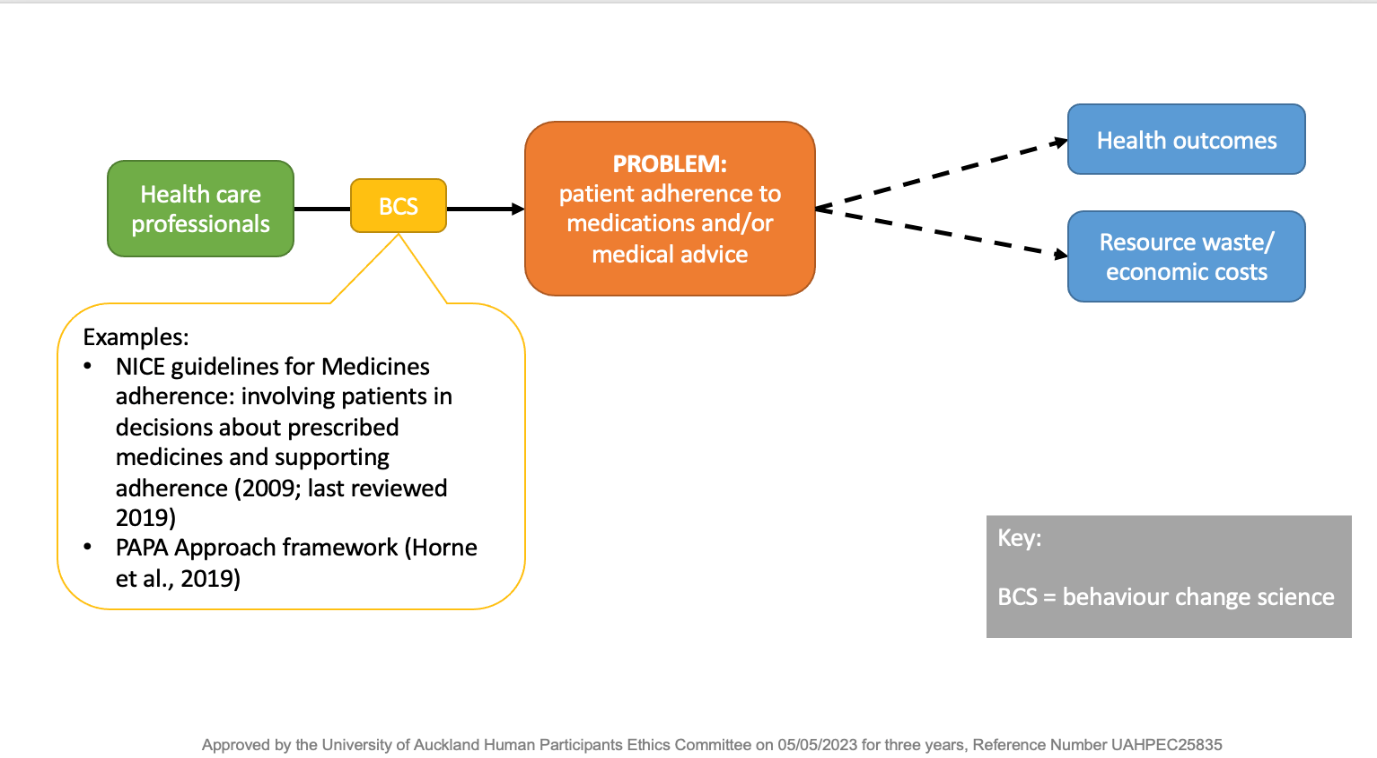


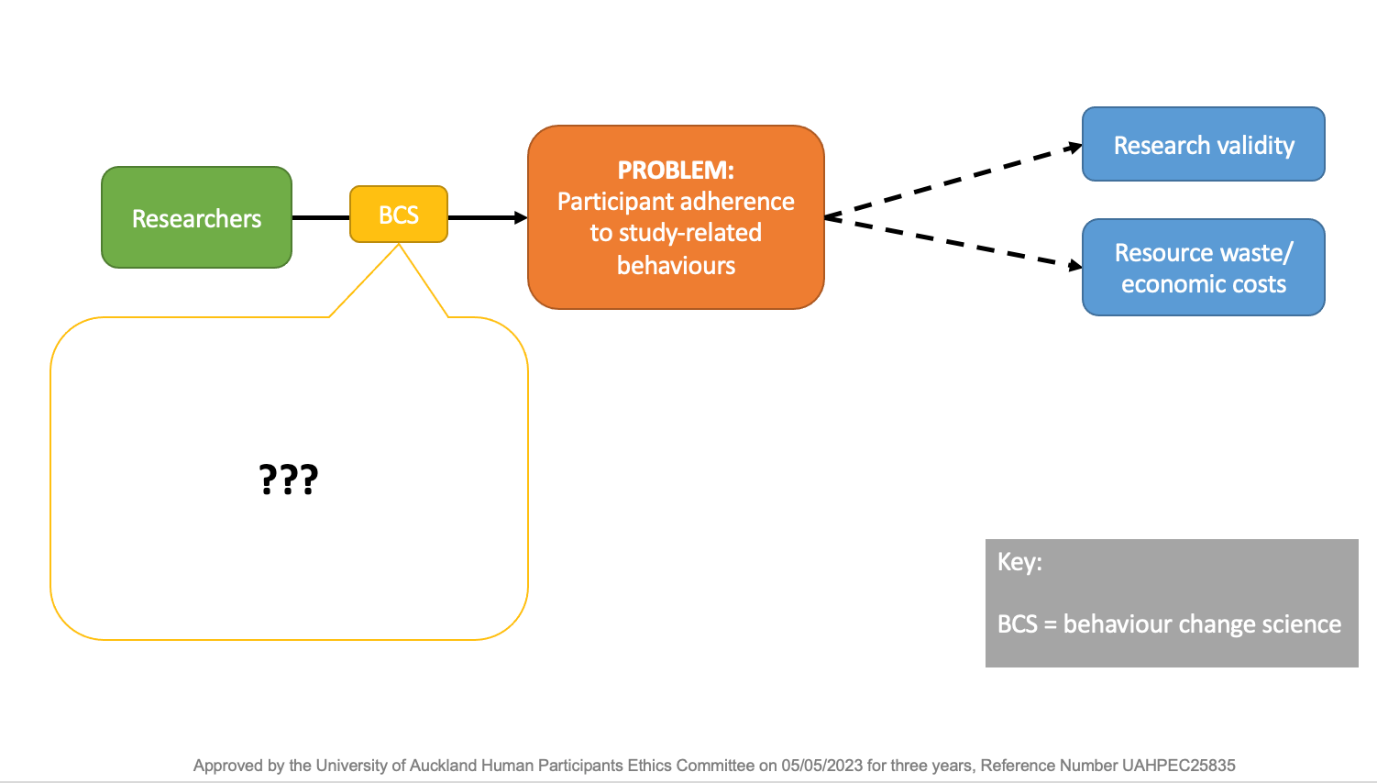


“You may be familiar with the problem of poor patient adherence to taking medications. This can lead to poor health outcomes, as well as resource waste. To combat this issue evidence suggests that using behaviour change science can help to improve adherence. Hence, frameworks making behaviour change science accessible and useable by healthcare professionals (HCP) have been developed. These frameworks can be used by HCP to improve patient adherence to medications. We know that participant adherence to study-related behaviours within trials can also be poor. In particular, adherence to dietary behaviours in nutrition trials can be a challenge. This can impact research validity and lead to resource waste. Similar to patient adherence to taking medications, it is proposed that behaviour change science can be systematically used by researchers to improve participant adherence within nutrition trials. What this exactly looks like is not known. For this next section of the interview, we are wanting to explore your thoughts on this proposal.”

1. *Presseau, J., McCleary, N., Lorencatto, F., Patey, A. M., Grimshaw, J. M., & Francis, J. J. (2019). Action, actor, context, target, time (AACTT): a framework for specifying behaviour. Implementation Science, 14(1), 102. https://doi.org/10.1186/s13012-019-0951-x* [↑](#footnote-ref-2)
2. *Rachel Davis, Rona Campbell, Zoe Hildon, Lorna Hobbs, Susan Michie. (2014). Theories of behaviour and behaviour change across the social and behavioural sciences: a scoping review. Health Psychology Review, vol. 9 (3), 323-344.* [↑](#footnote-ref-3)
