# Supplementary material for: Exploring how researchers consider nutrition trial design and participant adherence: a theory-based analysis
Source: Front Nutr. 2024 Dec 17;11:1457708. doi: 10.3389/fnut.2024.1457708 (PMC11685074; doi:10.3389/fnut.2024.1457708)
Supplement: Supplementary file 3 [file Supplementary_file_3.docx]

**TDF Coding Guideline for Interview Script**

*Based on Coding Guidelines developed by Hanrahan et al. (2022).*

**Part 1.** Understand the determinants using the TDF that influence how researchers design nutrition trial components to support participant adherence to dietary behaviours.

**Coding instructions for Part 1 of Interview**

**Behaviour of interest:** how researchers design and conduct human trials to promote/enhance participant adherence to dietary behaviours required or desired within that trial.

**AACTT Specification**

- Action: Any action taken, implicitly or explicitly, by researchers to encourage participant adherence to dietary behaviours
- Actor: Researchers involved in the design of human trials that involve participants performing a dietary behaviour
- Context: Tertiary education, workplaces and other settings that conduct human trials
- Target: Trial participants
- Time: During the design and conduct of human (nutrition) trials

1. The framework used for data analysis is the 14-Domain Theoretical Domains Framework.
2. Code all relevant text into each domain (i.e. provide context to demonstrate how the text is associated with a domain), but also highlight the specific sections of the text within that are specifically related to the domain. Wherever possible use its corresponding constructs to justify coding into the domain (definitions of constructs and domains are included below).
3. Please use the “Decision Rule for behaviour” column to supplement the description of the domains and constructs for this context. Also see the decision rule column for information regarding text that could be justifiably coded into multiple domains.

| **TDF Domain** | **Construct** | **Decision Rule for behaviour** |
| --- | --- | --- |
| **1. Knowledge**  An awareness of the existence of something | *Knowledge* (including knowledge of condition/scientific rationale): *An awareness of the existence of something*  *Procedural knowledge: knowing how to do something.*  Knowledge of task environment: *Knowledge of the social and material context in which a task is undertaken.* | **Appropriate coding to this domain:**  Knowledge or lack of knowledge of;   - Dietary changes being behaviours - Level of participant adherence to these behaviours within their trial - How adherence was measured in their trial   **Inappropriate coding to this domain:**   - The active ‘measuring’ of adherence (code to *Skills*) |
| **2. Skills**  An ability or proficiency acquired through practice | Skills: *An ability or proficiency acquired through training and/or practice.*  Skills development: *The gradual acquisition or advancement through progressive stages of an ability or proficiency acquired through training and practice.*  Competence: *One’s repertoire of skills, and ability especially as it is applied to a task or set of tasks.*  Ability: *Competence or capacity to perform a physical or mental act. Ability may be either unlearned or acquired by education and practice.*  Interpersonal skills: *An aptitude enabling a person to carry on effective relationships with others, such as an ability to cooperate, to assume appropriate social responsibilities or to exhibit adequate flexibility.*  Practice: *Repetition of an act, behaviour, or series of activities, often to improve performance or acquire a skill.*  Skills assessment: *A judgement of the quality, worth, importance. Level or value of an ability or proficiency acquired through training and practice.* | **Appropriate coding to this domain:**   - Development or use of strategies for improving participant adherence to dietary behaviours within the trial - Competence and ability in measuring adherence to dietary behaviours   **Inappropriate coding to this domain:**   - How researchers feel about their current skill level (code to *Beliefs about capabilities* or *Emotion*) - Strategies used to enhance adherence to non-dietary behaviours |
| **3. Social/**  **Professional Role and Identity**  A coherent set of behaviours and displayed personal qualities of an individual in a social or work setting. | Professional identity: *The characteristics by which an individual is recognised relating to, connected with or befitting a particular profession.*  Professional role: *The behaviour considered appropriate for a particular kind of work or social position.*  Social identity: *The set of behavioural or personal characteristics by which an individual is recognizable [and portrays] as a member of a social group*.  Identity: *An individual’s sense of self defined by a) a set of physical and psychological characteristics that is not wholly shared with any other person and b) a range of social and interpersonal affiliations (e.g., ethnicity) and*  *social roles.*  Professional boundaries: *The bounds or limits relating to, or connected with a particular profession or calling.*  Professional confidence: *an individual’s belief in his or her repertoire of skills and ability especially as it is applied to a task or set of tasks.*  Group identity: *the set of behavioural or*  *personal characteristics by which an individual is recognizable [and portrays] as a member of a group.*  Leadership: *The processes involved in leading others, including organising, directing, coordinating and motivating their efforts toward achievement of certain group or organization goals.*  Organisational commitment: *An employee’s dedication to an organisation and wish to remain part of it. Organisational commitment is often*  *described as having both an emotional or moral element and a more prudent element.* | **Appropriate coding to this domain:**   - What researchers believe their role to be in relation to enhancing participant adherence in trial design - Link between profession/role in trial and concern of participant adherence to dietary behaviours   **Inappropriate coding to this domain:**   - If researchers describe relationship i.e. a need for someone else’s behaviour (code at *Social Influences)* - Identity of a organisational culture/climate i.e. an institution having a reputation of using behaviour change science (code at *Environmental Context and Resources*) |
| **4. Beliefs About Capabilities**  Acceptance of the truth, reality, or validity about an ability, talent or facility that a person can put to constructive use | Self-confidence: *Self-assurance or trust in one’s own abilities, capabilities and judgement.*  Perceived competence: *An individual’s belief in her or her ability to learn and execute skills.*  Self-efficacy: *An individual’s capacity to act effectively to bring about desired results, as perceived by the individual.*  Perceived behavioural control*:* *an individual’s perception of the ease or difficulty of performing the behaviour of interest.*  Beliefs: The thing believed*; the proposition or set of propositions held true.*  Self-esteem: *The degree to which the qualities and characteristics contained in one’s self concept are perceived to be positive.*  Empowerment*:* *The promotion of the skills, knowledge and confidence necessary to take great control of one’s life as in certain educational or social schemes; the delegation of increase decision-making powers to individuals or groups in a society or organization.*  Professional confidence: *An individual’s beliefs in his or her repertoire of skills, and ability, especially as it is applied to a task or set of tasks.* | **Appropriate coding to this domain:**   - Researchers’ confidence in ability to design/conduct a nutrition study with high adherence to dietary behaviours - How easy or difficult researchers’ feel it is to enhance participant adherence - Self-efficacy and beliefs regarding utilising behaviour change science in trial design   **Inappropriate coding to this domain:**   - Expectations about participant adherence (code to *Beliefs about Consequences*) - Expectations of outcomes of strategies used to enhance participant adherence (code to *Beliefs about Consequences*) |
| **5. Optimism**  The confidence that things will happen for the best or that desired goals will be attained | Optimism: *The attitude that outcomes will be positive and that people’s wishes or aims will be ultimately fulfilled.*  Pessimism: *The attitude that things will go wrong and that people’s wishes or aims are unlikely to be fulfilled.*  Unrealistic optimism: *the inert tendency for humans to over-rate their own abilities and chances of positive outcomes compared to those of other people.* | Researchers’ descriptions of their level of optimism regarding the ability to design a trial that enables and encourages participants to adhere (optimistic for enhancing adherence of participants); Both positive and negative comments  **Appropriate coding to this domain:**   - Researchers’ discussion of optimism or pessimism related to the design of a trial impacting participant adherence   **Inappropriate coding to this domain:**   - Feeling of anxiety, stress or burnout (code to Emotion) - Positive and negative outcomes of using strategies in trial design to enhance participant adherence (code to *Beliefs about consequences*) |
| 1. **6. Beliefs about consequences**   Acceptance of the truth, reality or validity about outcomes of a behaviour in a given situation. | Beliefs: *The thing believed; the proposition or set of propositions held true.*  Outcome expectancies: *Cognitive, emotional, behavioural, and affective outcomes that are assumed to be associated with future or intended behaviour. These assumed outcomes can either promote or inhibit future behaviours.*  Characteristics of outcome expectancies: *Characteristics of the cognitive, emotional and behavioural outcomes that individuals believe are associated with future or intended behaviours and that are believed to either promote or inhibit these behaviours. These include whether they are sanctions/rewards,*  *proximal/distal, valued/not valued,*  *probable/improbable. Salient/not salient, perceived risks or threat*s.  Anticipated regret: *A sense of the potential negative consequences of a decision that influences the choice made: for example an individual may decide not to make an investment because of the feelings associated with an imagined loss*  Consequents: An outcome behaviour in a given situation | Positive or negative expectancies of designing and conducting trials to enhance adherence to dietary behaviours within trials.  **Appropriate coding to this domain:**   - Descriptions of how trial design impacts participant adherence - Conduct behaviours within trial to promote adherence   **Inappropriate coding to this domain:**   - Beliefs about whether researchers can impact participant adherence to dietary behaviours through trial design (code to *Beliefs about Capabilities*) - The confidence that goals will be achieved (Code to *Optimism*) - Outcomes of high or low adherence within the study (Code to *Reinforcement*) |
| **7. Reinforcement**  Increasing the probability of a response by arranging a dependent relationship, or contingency, between the response and a given stimulus | Rewards (proximal/distal, valued/ not valued, probable/improbable): *Return or recompense made to, or received by a person contingent on some performance.*  Incentives: *An external stimulus, such as condition or object, that enhances or serves as a motive for behaviour.*  Punishment: *The process in which the relationship between as response and some stimulus or circumstance results in the response becoming less probable; a painful, unwanted or undesired event or circumstance imposed as a penalty on a wrongdoer.*  Consequents: *An outcome of behaviour in a given situation.*  Reinforcement: *A process in which the frequency of a response is increased by a dependent relationship or contingency with a stimulus*  Contingencies: *A conditional probabilistic relation between two events. Contingencies may be arranged via dependencies or they may emerge by accident.*  Sanctions: *A punishment or other coercive measure, usually administered by a recognized authority, that is used to penalise and deter inappropriate or unauthorized actions.* | **Appropriate coding to this domain:**   - Rewards or incentives for enhancing participant adherence in trials - Non-material reward - Social reward, intrinsic reward - Material rewards - Perceived punishments, consequents, reinforcements, contingencies, sanctions related to having different levels of participant adherence to dietary behaviours   **Inappropriate coding to this domain:**   - Opportunities to reinforce or consolidate skills in trial design to enhance adherence (code to *Skills* - Construct: Practice/Skill development) |
| **8. Intentions**  A conscious decision to perform a behaviour or a resolve to act in a certain way | Stability of intentions: *ability of one’s resolve to remain in spite of disturbing influences.*  Stages of Change model: *A model that proposes that behaviour change is accomplished through five specific stages.*  Transtheoretical model and stages of change: *a five-stage theory to explain changes in people’s health behaviour. It suggests that change takes time, that different interventions are effective at different stages, and that there are multiple outcomes occurring across the stages.* | **Appropriate to code in this domain:**   - Researcher’s descriptions of how motivated they are to have high participant adherence to dietary behaviours in trials - Researcher’s descriptions of when they are and are not inclined to attempt to enhance participant adherence (could also code to other domains as necessary i.e. *Beliefs about Consequences*) Note: Indicator of intention must be explicit and not inferred - Can also include behaviours related to participant adherence such as measuring it within the study   **Inappropriate coding to this domain:**   - This is different from how much of a priority enhancing adherence is in trial design is to them (code under *Goals*)   Be careful not to code the reasons for the intention (focus on statements that directly reflect their intention and motivation). |
| **9. Goals**  Mental representations of outcomes or end states that an individual wants to achieve | Goals (distal/proximal): *Desired state of affairs of a person or system, these may be closer (proximal) or further away (distal).*  Goal priority: *Order of importance or urgency of end state toward which one is striving.*  Goal/target setting: *A process that establishes specific time based behavioural targets that are measurable, achievable and realistic.*  Goals (autonomous/controlled): *The end state toward which one is striving: the purpose of an activity or endeavour. It can be identified by observing that a person ceases or changes their behaviour upon attaining this state; proficiency in a task to be achieved within a set period of time.*  Action planning: *The action or process of forming a plan regarding a thing to be done or a deed.*  Implementation intention: *The plan that one creates in advance of when, where an how one will enact a behaviour.* | **Appropriate coding to this domain:**   - Comments related to goal setting: descriptions of specific goals for enhancing participant adherence to dietary behaviours - Goal priority, action planning and implementation intention related to enhancing participant adherence - Descriptions that highlight whether considering participant adherence in trial design was a priority for the researchers - Practical plans to enhancing participant adherence in trial design or not   **Inappropriate coding to this domain:** |
| 1. **10. Memory, attention and decision processes**   The ability to retain information, focus selectively on aspects of the environment and choose between two or more alternatives | Memory: *The ability to retain information or a representation of a past experience, based on the mental processes of learning or encoding retention across some interval of time, and retrieval or reactivation of the memory; specific information of a specific task.*  Attention: *A state of awareness in which the senses are focussed selectively on aspects of the environment and the central nervous system is in a state of readiness to respond to stimuli.*  Attention control: *The extent to which a person can concentrate on relevant cues and ignore all irrelevant cues in a given situation.*  Decision making: *The cognitive process of choosing between two or more alternatives, ranging from the relatively clear-cut to the complex.*  Cognitive overload/tiredness: *The situation in which the demands placed on a person by mental work are greater than a person’s mental abilities.* | **Appropriate coding to this domain:**   - Comments relating to when/why it would be easy to forget about participant adherence to dietary behaviours in trial design - Descriptions of decision processes regarding using strategies and associated tools in trial design to enhance participant adherence - Cognitive overload/fatigue related to enhancing participant adherence in trial design and conduct   **Inappropriate coding to this domain:**   - If researchers simply indicate their general awareness of participant adherence, without citing how this influences decision-making processes (code under *Knowledge*) - Discussion of system pressures that impact on decisions regarding trial design to enhance participant adherence (code to *Environmental Context and Resources*: e.g. Environmental Stressors) |
| **11. Environmental Context and Resources**  Any circumstance of a person’s situation or environment that discourages or encourages the development of skills and abilities, independence, social competence, and adaptive behaviour  *)* | Environmental stressors*: External factors in the environment that cause stress.*  Resources/material resources: C*ommodities and human resources used in enacting a behaviour.*  Organizational culture/climate: *A distinctive pattern of thought and behaviour shared by members of the same organization and reflected in their language, values, attitudes, beliefs and customs.*  Salient events/critical incidents: *Occurrences that one judges to be distinctive, prominent or otherwise significant.*  Person x environment interaction: *Interplay between the individual and their surroundings.*  Barriers and facilitators: *In psychological contexts, barriers/facilitators are mental, emotional or behavioural limitations/strengths in individuals or groups.* | **Appropriate coding to this domain:**   - Comments regarding the equipment required to enhance participant adherence in trial design - Comments regarding the setting in which trial design occurs - Comments regarding human resources required to design trials to enhance participant adherence - Organisational culture/climate, impacting on ability to design trials to support participant adherence - Description of how much time is required to design trials to enhance participant adherence   **Inappropriate coding to this domain:**   - If researchers advocate the need for training on how to design trials to support participant adherence (code under *Skills)* |
| **12. Social Influences**  Those interpersonal processes that can cause individuals to change their thoughts, feelings, or behaviours | Social pressure: *the exertion of influence on a person or group by another person or group.*  Social norms: *Socially determined consensual standards that indicate a) what behaviours are considered typical in a given context and b) what behaviours are considered proper in the context.*  Group conformity: *The act of consciously maintaining a certain degree of similarity to those in your general social circles.*  Social comparisons: *The process by which people evaluate their attitudes, abilities or performance relative to others.*  Group norms: *Any behaviour, belief, attitude or emotional reaction held to be correct or acceptable by a given group in society.*  Social support: *The apperception or provision of assistance or comfort to others, typically in order to help them cope with a variety of biological, psychological and social stressors. Support may arise from any interpersonal relationship in an individual’s social network, involving friends, neighbours, religious institutions, colleagues, caregivers of support groups.*  Power: *The capacity to influence others, even when they try to resist this influence.*  *Intergroup conflict: Disagreement or*  *confrontation between two or more groups and their members. This may involve physical violence, interpersonal discord, or psychological tension.*  Alienation: *Estrangement from one's social group; a deep seated sense of dissatisfaction with one's personal experiences that can be a source of lack of trust in one's social or physical environment or in oneself; the experience of separation between thoughts and feelings.*  Group identity: *the set of behavioural or personal characteristics by which an individual is recognizable [and portrays] as a member of a group.*  Modelling: *In developmental psychology the process in which one or more individuals or other entities serve as examples (models) that a child will copy.* | **Appropriate coding to this domain:**   - Discussion about how other people influence decisions about trial design. Includes influence of other researchers/clinical colleagues/trial staff/stakeholders - Comments relating to the actions of others and how this influences decisions to design trials to enhance participant adherence - Discussing importance of others engagement/buy-in - Social pressure to design trials to enhance participant adherence or not - Discussion of team roles/dynamics within the trial design environment and how this influences their own behaviour (e.g. “these people do this, so I do this…”)   **Inappropriate coding in this domain:**   - Comments regarding researcher’s own level of equipoise; this may be coded under “*Beliefs about consequences*” if applicable - Specific descriptions of the roles of others; this should be coded under “*Social Professional Role and Identity*” - Comments regarding workplace without mention of personal; this should be coded to *“Environmental context and resources”* |
| **13. Emotion**  A complex reaction pattern, involving experiential, behavioural and physiological elements, by which the individual attempts to deal with a personally significant matter or event. | Fear: *An intense emotion aroused by the detection of imminent threat, involving an immediate alarm reaction that mobilizes the organism by triggering a set of physiological changes.*  Anxiety: *A mood state characterized by apprehension and somatic symptoms of tension in which an individual anticipates impending danger, catastrophe or misfortune.*  Affect: *An experience or feeling of emotion, ranging from suffering to elation, from the simplest to the most complex sensations of feelings, and from the most normal to the most pathological emotional reactions.*  Stress: *A state of physiological or psychological response to internal or external stressors.*  Depression: *A mental state that presents with depressed mood, loss of interest or pleasure, feelings of guilt or low self-worth, disturbed sleep or appetite, low energy, and poor concentration.*  Positive/negative affect: *the internal feeling/state that occurs when a goal has/has not been attained. A source of threat has/has not been avoided, or the individual is/is not satisfied with the present state of affairs.*  Burn-out: *Physical, emotional or mental exhaustion, especially in one’s job or career, accompanied by decreased motivation, lowered performance and negative attitudes towards oneself and others.* | Includes comments about emotional experiences (actual or preconceived) that (may) influence decisions to use behaviour change science in trial design (could also be coded under *Memory, Attention & Decision Processes*).  **Appropriate coding to this domain:**   - Descriptions of emotions experienced by researchers (could also be preconceived) before/during/after trial design regarding participant adherence - Description of when researchers would be worried/concerned about participant adherence in trials   **Inappropriate coding to this domain:**   - Description of trial participants emotions regarding adherence to dietary behaviours (code to *Social Influences*) |
| **14. Behaviour regulation**  Anything aimed at managing or changing objectively measured actions | Self-monitoring: *A method used in behavioural management in which individuals keep a record of their behaviour, especially in connection with efforts to changes or regulate the self; a personality trait reflecting an ability to modify one’s behaviour in response to a situation.*  Breaking habit: *to discontinue a behaviour or sequence of behaviours that is automatically activated by relevant situational cues.*  Action planning: *The action or process of forming a plan regarding a thing to be done or a deed.* | **Appropriate coding to this domain:**   - Description(s) of a process(es) that would remind researchers to use strategies to enhance participant adherence in trial design - Discussion regarding habits and breaking old habits to facilitate using designing trials to enhance participant adherence - Description of self-regulatory strategies that influence trial design |

**Coding instructions for Part 2 of Interview**

1. Read transcripts
2. Code sentences deductively to 6x COM domains
3. Read through each COM domain to identify beliefs (form into statements)
4. Organise belief statements into themes

**Behaviour of interest:** Researchers explicitly using behaviour change science (e.g. models, theories and frameworks) when designing trials to enhance participant adherence to dietary behaviour changes required/desired within the trial.

| **COM-B Domain** | **Related TDF domain** | **Decision Rule for behaviour** |
| --- | --- | --- |
| **1. Psychological capability** | Knowledge, behaviour regulation, memory, attention and decision making | **Appropriate coding to this domain:**  Knowledge or lack of knowledge of behaviour change science  **Inappropriate coding to this domain:**  The active using of behaviour change science (code to *Skills*) |
| **2. Physical capability** | The ability and skills to do something. | **Appropriate coding to this domain:**  Skills and/or ability to use behaviour change science when designing trials  **Inappropriate coding to this domain:**  Ability to use behaviour change or not dependent on physical resources (*code to physical opportunity)* |
| **3. Social opportunity** | Social influences | **Appropriate coding to this domain:**  Comments regarding how other people (stakeholders, participants, other researchers) influence their decision/ability to use behaviour change science when designing studies.  **Inappropriate coding to this domain:**  Researchers’ own beliefs about the consequences of performing this behaviour (code to *reflective motivation)* |
| **4. Physical opportunity** | Environmental context and resources | **Appropriate coding to this domain:**  Barriers and enablers to using behaviour change science in trial design regarding physical resources and environmental context  **Inappropriate coding to this domain:**  A researchers own physical capability (code to *skills)* |
| **5. Automatic motivation** | Reinforcement, emotions | **Appropriate coding to this domain:**  Feelings that influence decision to use behaviour change science when designing trials (or not)  Situations or outcomes that encourage or discourage researchers from using behaviour change  **Inappropriate coding to this domain:**  Beliefs about what the consequences or outcomes will be (code to *reflective motivation)* |
| **5. Reflective motivation** | Intention, goals, social/professional role and identity, beliefs about capabilities, belief about consequences, optimism | **Appropriate coding to this domain:**  Conscious level of motivation and things influencing this such as beliefs about role, consequences, capabilities  Reported confidence in using behaviour change science  **Inappropriate coding to this domain:**  Actual capability (as opposed to perceived) (code to *skills)* |
