# Supplementary material for: Exploring how researchers consider nutrition trial design and participant adherence: a theory-based analysis
Source: Front Nutr. 2024 Dec 17;11:1457708. doi: 10.3389/fnut.2024.1457708 (PMC11685074; doi:10.3389/fnut.2024.1457708)
Supplement: Supplementary file 4 [file Supplementary_file_4.docx]

Supplementary file 4

Table 1. Behaviour change techniques interviewees reported using in their trial to promote participant adherence to dietary behaviours.

| **Behaviour change techniques** |
| --- |
| 1.2 Problem solving |
| 1.3 Goal setting (outcome) |
| 1.7 Review outcome goal(s) |
| 1.8 Behavioural contract |
| 2.1 Monitoring of behaviour by others without feedback |
| 2.3 Self-monitoring of behaviour |
| 3.1 Social support (unspecified) |
| 4.1 Instruction on how to perform the behaviour |
| 5.1 Information about health consequences |
| 7.1 Prompts/cues |
| 9.1 Credible source |
| 10.1 Material incentive (behaviour) |
| 12.5 Adding objects to the environment |
| 14.2 Punishment |
