# Supplementary material for: Exploring how researchers consider nutrition trial design and participant adherence: a theory-based analysis
Source: Front Nutr. 2024 Dec 17;11:1457708. doi: 10.3389/fnut.2024.1457708 (PMC11685074; doi:10.3389/fnut.2024.1457708)
Supplement: Supplementary file 5 [file Supplementary_file_5.docx]

**Supplementary file 5.**

**Table 1.** Researchers’ perceived barriers and enablers to using behaviour change science in the design of nutrition trials to support participant adherence.

| COM-B Domain | Summary | Quotes |
| --- | --- | --- |
| Capability (psychological) | Many interviewees expressed not having the knowledge regarding what or how to use behaviour change science. Additionally, one behaviour change expert expressed concern at researchers using behavioural science when it’s outside their area of expertise. | “I don’t think I am that confident that I would have the knowledge unless it was like, very well advertised, you know like every time I go to a conference…” P3  “…no one explained us how to do that, so I'm not really confident that I'm doing the best things.” P5  “I feel like others view behavioural science as a very soft and easy thing to do and that running a behavioural trial would be super easy and straightforward, and that you know, regardless of your degree, that you would be able to do it when I feel like behavioural scientists wouldn't barrel forward and prescribe medicines, or, you know, engage in other people's areas of expertise. But that's often done by people outside of behavioural sciences.” P1 |
| Capability (physical) | Interviewees reported a range of confidence levels in their perceived ability to be able to use behaviour change science in the design of trials. This often depended on their area of expertise and years of experience designing trials.  Suggestions to increase confidence in their skills included further training in applying behavioural science and having real life examples to work from. Many were confident in their skills to do so if trainings were provided. | “It’s not something that I’ve ever been, I’ve ever studied, or have much experience in doing. I’m sure if we came up with something and I had some training or some expertise, then I could manage” P11  “I think, with my background as a dietitian I've learned about it, behaviour change and motivational interviewing during undergrad. And I have those foundational skills.” P4  “…I think the best way for people like me to be able to use something like that would be really to kind of have an easy, like I said, cheat sheet that you know what you need to do and it’s simple to follow.” P3 |
| Opportunity (social) | In general, interviewees responded positively about the acceptance of using behavioural science amongst them and their colleagues. Two interviewees were sceptical that it would be encouraged by other researchers and reviewers.  Additionally, a key enabler identified through the interviews for using behaviour change science was collaboration and networking with colleagues, particularly those who have more experience in implementing it; talking with colleagues was perceived to enable researchers to question and improve current practices. | “I think I think people probably would support, especially if you can show that it does help with adherence.” P3  “I think, because I work with a lot of psychologists, it would be, positively viewed. I think a lot of the old, old school nutritionists may question it. But I think, it would be viewed favourably amongst most people.” P10  Interviewer: “to what extent, do you feel this would be accepted or supported by the people around you, such as colleagues and stakeholders?”  P5: “Not so much. And in general, colleagues, physicians, and researchers are not really interested in this aspect… they think only to cure and treat, and not to listen to subjects…”  “I feel like what often gives me those ideas is talking with a colleague sometimes about a grant idea or just a project that they're working on, and realizing that there are differences in the way that a standard intervention is implemented, and starting to question the way that I've been taught to do things because I think that often we don't question some of these non-evidence-based practices, and we just blindly move ahead, as you know, “Oh, well, you know, weekly sessions are the way to go”.” P1  “You would need someone who is used to doing it to advise you and or help design it and potentially implement it… cause it’s an area outside of my own expertise.” P11 |
| Opportunity (physical) | Interviewees perceived limited time and funding would stop them using behaviour change science in trial design.  Additionally, access to resources, trainings, expertise. and literature were seen as an enablers, as these are perceived to increase capability. | Interviewer: “what would stop researchers from using behaviour change science in this way?”  P10: “If they didn't get funding from it.”  “I think what I see as a challenge in research is that you don't have that kind of timeframe” P11  “if it took a lot of extra time or if I had to spend thousands of dollars to be trained in a particular method, then that would definitely hold me back” P7  “Not resources in terms of money because I don't think we need a lot of money to do that just interest, time, and someone who can teach us.” P5 |
| Motivation (automatic) | Some interviewees reported they would be motivated to use it if there was reinforcement from funding agencies.  Equally, some interviewees expressed how they hadn’t thought about using it; it doesn’t exist as a habit for them. | “if the funding agency was pushing it, of there was a grant that said, you know, we’d like to see if we could do this dietary behaviour… then I’d want to use it.” P2  “It's not really something I've really thought about, but it's interesting to think about it in the context of making people adhere to research in the same way that we also try and make people change in clinical practice, and it's difficult enough to do it in clinical practice.” P11 |
| Motivation (reflective) | About half of the interviewees reported high motivation to use behavioural science in this context as they saw it as important for improving trial design and validity, and beneficial for the wellbeing of their participants.  The other half of participants expressed lower motivation around using it, primarily without existing evidence that it would produce greater adherence than their current practice. | “…very motivated. I definitely, you know, has already seen the value of including it in our designs” P7  “I think every clinical trial should have some sort of behaviour change science in it, because it's so important because you need to learn about the person and how they think.” P4  “I think if they believe that it will work, they would use it... they probably don't believe the evidence. I’m not convinced the evidence is very strong.” P2  “Of course, I would want to see that it really works first.” P3  “That’s the first barrier, I think, is just not having the science there to support doing it this way” P6 |
